# NMDA receptor-dependent long-term depression in the lateral habenula: implications in physiology and depression

**DOI:** 10.1038/s41598-020-74496-w

**Published:** 2020-10-21

**Authors:** Miseon Kang, Jihyun Noh, Jun-mo Chung

**Affiliations:** 1 Department of Brain and Cognitive Sciences, Brain Disease Research Institute, Ewha Womans University, Seoul, Republic of Korea; 2 Department of Science Education, College of Education, Dankook University, Yongin-si, Republic of Korea; 3 Emotion, cognition & behavior research group, Korea Brain Research Institute (KBRI), 61, Cheomdan-ro, Dong-gu, Daegu, 41062 South Korea

**Keywords:** Neuroscience, Physiology

## Abstract

Abnormally increased neuronal activity in the lateral habenula (LHb) is closely associated with depressive-like behavior. Despite the emphasis on the pathological importance of NMDA receptor (NMDAR)-dependent long-term depression (LTD) and the involvement of calcium permeable AMPA receptor (CP-AMPAR) as major Ca^2+^ source, the functions of NMDAR and CP-AMPAR on LTD modulation in the LHb still have not been fully investigated. Here, we found that NMDAR-dependent LTD by low frequency stimulation was induced in both synaptic and extrasynaptic regions in the LHb. In addition, CP-AMPAR was necessary for the activation of NMDAR in the induction phase of NMDAR-dependent LTD. The acute stress, which induced depressive behavior, had a blocked effect on synaptic NMDAR-dependent LTD but left extrasynaptic NMDAR-dependent LTD intact. These findings show that NMDAR-dependent LTD in LHb plays an important role in regulating neuronal activity, which is probable to be excessively increased by repeated stress, via maintaining homeostasis in both synaptic and extrasynaptic regions of the LHb. Moreover, NMDAR and CP-AMPAR may serve as a depression-related modulator and be regarded as a promising therapeutic target for treatment of psychopathology such as depression.

## Introduction

Long-lasting suppression of glutamatergic transmission is linked with the homeostasis of basal synaptic transmission by the regulation of neuronal output transmitted into the monoaminergic system in the lateral habenula (LHb)^1^. Several studies have reported that alteration of long-term depression (LTD) in the LHb is an important molecular mechanism to understand psychopathological changes such as depression and addiction^2,3^. Low-frequency stimulation (LFS)-induced LTD in the LHb is dependently modulated by the activation of group I metabotropic glutamate receptor (mGluR)^1,3^. The mGluR-dependent LTD modulated the excitatory and inhibitory output signaling, and consequently, it engaged in modulating the neuronal activity in the LHb. Although calcium-permeable AMPA receptor (CP-AMPAR), considered to be a Ca^2+^ source, is involved in changing LTD to long-term potentiation (LTP) in the LHb neuron^1,4^, a molecular mechanism concerning the fundamental modulation of LTD has not been fully reported. It is generally believed that mGluR-dependent LTD causes CP-AMPAR to change into calcium-impermeable (CI)-AMPAR^5–7^. However, mGluR-dependent LTD is independently modulated without having an effect on CP-AMPAR expression^1^. An electrophysiological investigation concerning the generation of LTD in the LHb should be undertaken to clarify the functional roles of CP-AMPAR in modulating LTD^8^.

A small component of NMDA receptor-mediated excitatory postsynaptic current is shown in the excitatory synapse of the rat LHb^9^. Until now, a functional investigation concerning the modulation of NMDAR-mediated LTD in the LHb was excluded because the NMDAR-mediated responses are minimal during the confirmation process of the current–voltage (I–V) curve through voltage clamp recordings^1,3,10^. However, the functional roles of the NMDAR-mediated LTD should be considered as well based on the fact that a significant component of NMDAR-mediated excitatory postsynaptic potential is shown in the mouse LHb synapse^1^ and levels of *NR1* and *NR2B* gene expression are significant in the rat LHb^11,12^. In general, the GluN2A and GluN2B subunits of NMDAR are key factors in determining the kinetics of NMDAR^13^. In addition, GluN2A is preferentially expressed at the synapse^14^, whereas GluN2B is expressed at both the synapse and extrasynapse^13–15^. Therefore, further investigation is required not only to confirm whether LTD induction is NMDAR-dependent but also to identify where NMDAR is located among the synaptic or extrasynaptic sites.

The LHb is a core brain region associated with depression^3,11,16,17^. In learned helplessness models, LHb neurons are found to be excessively activated^9,16^. In addition, mGluR-dependent LTD in the LHb is impaired by stress, and these synaptic alterations are found to be associated with depressive-like behavior as well^3^. A recent study reported that depressive-like behavior caused by repetitive stress is associated with an increase of bursting firing in the LHb, and inhibition of NMDAR by ketamine leads to an antidepressant effect^17^. However, in this report, ketamine has shown antidepressant effects by inhibiting NMDAR-dependent bursting, and there is still no research on the association between NMDA-dependent LTD in LHb and depressive behavior. In other words, we need to predict the functional role of NMDAR by examining the effect of NMDAR-dependent LTD on the LHb in the depression model.

In this study, we investigated overall properties of LFS-induced LTD and confirmed that the LFS-induced LTD was modulated by the activation of GluN2A- and GluN2B-containing NMDARs. In addition, we predicted the physiological and pathological function of NMDAR by determining NMDAR-dependent glutamatergic transmission in the synaptic and extrasynaptic regions of the LHb in normal and stressed rats.

## Materials and methods

### Animals

All animal experiments were performed in accordance with the guidelines of the Institutional Animal Care and Use Committee (IACUC) of Ewha Womans University (EWU) and carried out in accordance with local guidelines and regulations. This study was approved by the IACUC committee at Ewha Womans University (IACUC 18-010). Male Sprague Dawley (SD) rats (aged 17–24 days, weighing 20–35 g) were purchased from Orient Bio Inc. (Seongnam, South Korea). The animal center maintained constant humidity of 60%, temperature of 20–22 °C, and air ventilation, with a 12 h light–dark cycle (lights on at 7:00 a.m.).

### Restraint stress (RS) model and forced swimming test (FST)

Based on the method reported in a previous study^18^, we had male SD rats in their postnatal days 17–24 implanted into a well-ventilated 50-ml polypropylene conical tube. The rat’s bodies were forcibly compressed in the tube to immobilize its legs and tails. This control process was conducted from 10 a.m. for 90 min a day (see Fig. 4a). The stressed rats were subsequently forced to swim for 6 min, and the immobility time was measured for 5 min (except for 1 min of adaptation time), based on Porsolt’s study^19^. The RS model was composed of the rats that displayed an immobility time of more than 100 s during FST.

### Corticosterone assay

To determine the concentration of corticosterone (CORT), Corticosterone HS EIA kit (Immunodiagnostic Systems Ltd, IDS Ltd; Boldon, United Kingdom) was used. Blood was collected from normal and experimental groups and blood was centrifuged at 3,000 rpm for 15 min in a centrifuge (Eppendorf centrifuge 5810R, Brinkmann Instruments Co., Westbury, NY) to obtain blood-serum only. The experimental procedure was based on the experimental guide of the corticosterone HS EIA kit.

### Slice preparation

The rats were anesthetized with isoflurane aspiration. Sagittal sections of LHb slices (400-µm thick) were cut in ice-cold sucrose solution containing (in mM): sucrose, 201; NaHCO_3_, 26; glucose, 10; KCl, 3; NaH_2_PO_4_, 1.25; MgCl_2_, 3; and CaCl_2_, 1 (saturated with 95% O_2_/5% CO_2_). They were then transferred to a holding chamber filled with artificial cerebrospinal fluid (aCSF), containing (in mM): NaCl, 126; NaHCO_3_, 26; glucose, 10; MgSO_4_, 1.3; NaH_2_PO_4_, 1.25; KCl, 3; and CaCl_2_, 2.4. Freshly cut slices were placed in an incubating chamber containing aCFS and recovered at 30–31 °C for 1 h. Slices were then maintained in aCSF at room temperature (21–23 °C) prior to recording. For recording, each slice was transferred into a recording chamber and continuously superfused with oxygenated aCSF at a flow rate of 2–3 ml/min and temperature of 30 ± 2 °C using an in-line heater (Warner Instruments, Hamden, CT, USA). In the low-Mg^2+^ aCSF experiment, only 1.3 mM MgSO_4_ was changed to 0.5 mM concentration. To enhance the low osmolality level, glucose was added.

### Extracellular recording

Field EPSPs (ƒEPSPs) were recorded in the LHb with aCSF-filled 1–2 MΩ glass pipettes using an A-M Systems microelectrode AC amplifier and A/D converter (Digidata 1322A, Molecular Devices, Sunnyvale, CA, USA). Current stimuli were delivered to a constant stimulus isolation unit (150-µs pulse duration) (World Precision Instruments, Inc., Sarasota, FL, USA) by a Master-8 pulse generator (AMPI, Jerusalem, Israel). The stimulation rate was 0.067 Hz for all ƒEPSP recording experiments. After obtaining a stable baseline for 15 min, LTD was induced by applying 900 pulses at 1 Hz (LFS) through a concentric bipolar electrode (FHC, Bowdoin, Me, USA; 125 µm/Rnd/25 µm Pt-lr) with the same stimulating intensity used during baseline. The initial slope of the response was used to assess changes in synaptic strength.

### Drugs

For drug application, the aCSF solution was switched to the drug solution for at least 15 min before the induction of LFS. _D_-2-amino-5-phosphonovalerate (_D_-AP5), (*S*)-3,5-Dihydroxyphenylglycine ((S)-3,5-DHPG)), _DL_-threo-β-benzyloxyaspartate (_DL_-TBOA), (*S*)-(+)-α-Amino-4-carboxy-2-methylbenzeneacetic acid (LY367385), (+)-5-methyl-10,11-dihydroxy-5H-dibenzo(a,d)cyclohepten-5,10-imine[(+)-MK-801],*N*-[3-[[4-[(3-Aminopropyl)amino]butyl]amino]propyl]-1-naphthaleneacetamide (NASPM) trihydrochloride, *N*-Methyl-D-aspartic acid (NMDA), (α*R*,β*S*)-α-(4-Hydroxyphenyl)-β-methyl-4-(phenylmethyl)-1-piperidinepropanol (Ro 25-6981) maleate, and (1R^*^,2S^*^)-erythro-2-(4-Benzylpiperidino)-1-(4-hydroxyphenyl)-1-propanol (Ifenprodil) hemitartrate were obtained from Tocris Cookson (Bristol, UK). Aminoacetic acid, Aminoethanoic acid (Glycine) and [[[(1S)-1-(4-Bromophenyl)ethyl]amino](1,2,3,4-tetrahydro-2,3-dioxo-5-quinoxalinyl)methyl] phosphonic acid (PEAQX, NVP-AAM 077) tetrasodium hydrate were purchased from Sigma Aldrich (St. Louis, MO).

### Statistical analyses

Recordings were filtered at 1 Hz and 5 kHz, digitized at 10 kHz, and analyzed using pClamp9 (Molecular Devices). The ƒEPSP slope was calculated as the change at 10–90% of the baseline. The mean change in ƒEPSP slope was normalized to each slice’s baseline period. The sweep was presented by averaging 10 consecutive ƒEPSPs. Data were analyzed using GraphPad Prism (GraphPad Prism 8.0, USA). Data are presented as mean ± S.E.M. Two-way analysis of variance (ANOVA) was performed for comparison of all continuous variables between groups with Sidak’s multiple comparisons. Static significance was determined using an unpaired Student’s *t*-tests for comparisons between two groups. One-way ANOVA was performed for comparison of all continuous variables between groups; when indicated, a Dunnett’s correction was performed for post-hoc comparisons within and between groups (*vs.* baseline). Probability values of *P* < 0.05 were considered to represent significant differences. A dose–response relationship curve was analyzed using GraphPad Prism 8.0., and the curve was fit by a nonlinear regression.

## Results

### Long-term modulation of glutamatergic transmission in the LHb

**Figure 1:**
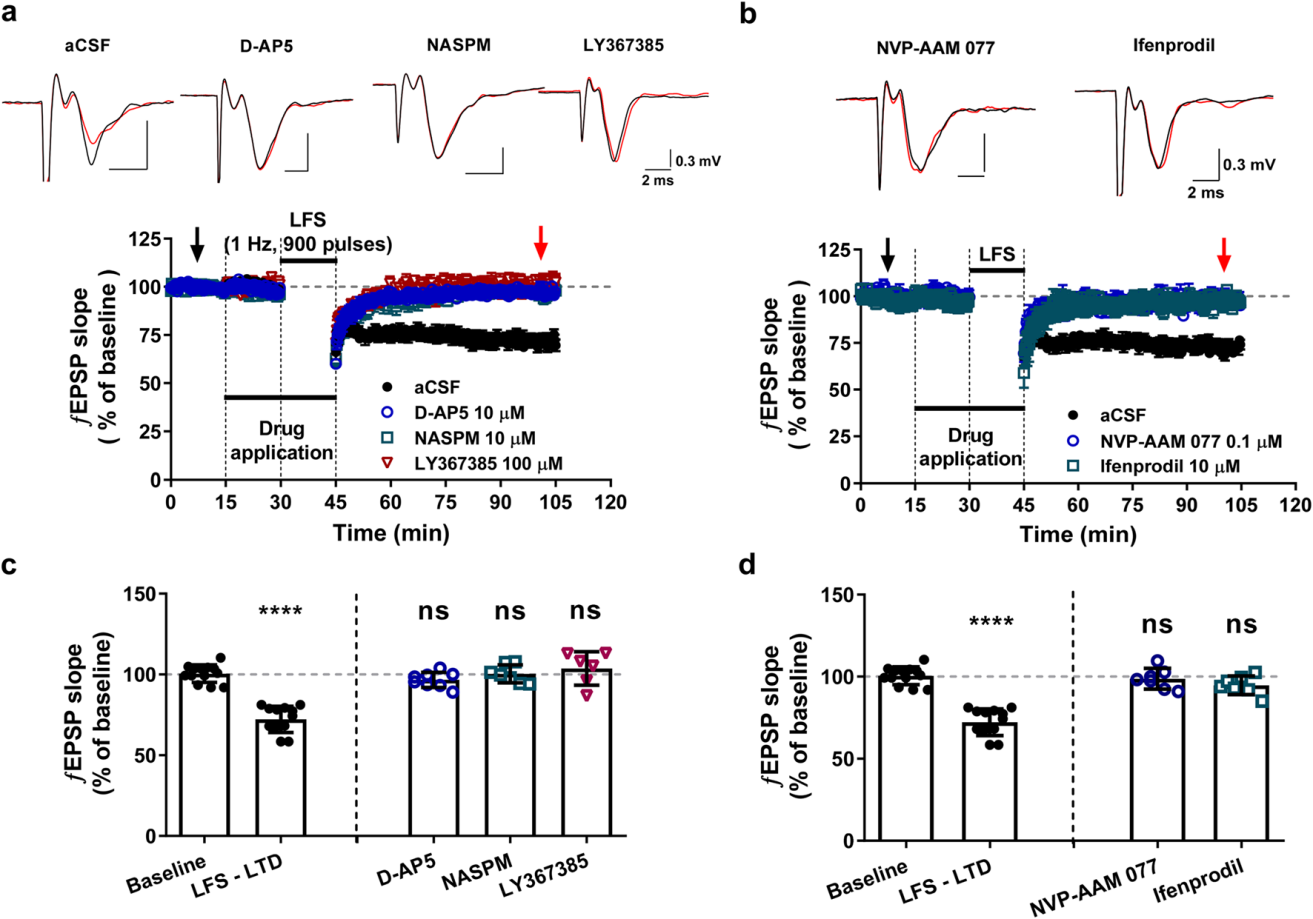
Determining NMDAR-dependent LTD in the LHb regions. (**a, b**) *Top*, The proposed ƒEPSP sweep shows the change in baseline (black trace, from black arrow in bottom) and after 55 min of LFS transmission (red trace, from red arrow in bottom). *Bottom*, The LFS (900 pulses at 1 Hz) elicited robust LTD. The regulation of LFS-induced LTD by selective glutamate receptor antagonists (**a**) and NMDAR subtype antagonists (**b**). (**c**) Summary histogram showing modulation of LTD by selective glutamate receptor antagonists (one-way ANOVA, F[4, 42] = 39.14; Dunnett’s post-hoc test as condition *vs*. baseline; LFS-LTD, 71.9 ± 4.2%, *n* = 10, ****P < 0.0001; D-AP5, 96.7 ± 1.7%, *n* = 8, ns, *P* = 0.5884; NASPM, 100.4 ± 2.1%, *n* = 7, ns, *P* > 0.9999; LY367385, 103.7 ± 4.2%, *n* = 6, ns, *P* = 0.7828). (**d**) Bar graph shows LTD is regulated by selective NMDAR subtype antagonist (one-way ANOVA, F[4, 42] = 37.97; Dunnett’s post-hoc test as condition *vs*. baseline; NVP-AAM 077, 99.1 ± 2.7%, *n* = 7, ns, *P* = 0.9533; ifenprodil, 97.4 ± 3.0%, *n* = 7, ns, *P* = 0.2148).

To determine which glutamate receptors are mainly involved in the long-term modulation of excitatory transmission in the LHb, we examined the effect of various glutamate receptor antagonists on LFS-induced LTD (Fig. 1). Basically, the changes in the slope of evoked ƒEPSPs was analyzed to avoid the interference of population spike in observing AMPA-mediated synaptic transmission (Fig. S1). The LFS (900 pulses each with a pulse duration of 150 μs at 1 Hz) did not induce the LTD at all under the presence of LY3677385 (100 μM) (Fig. 1a,c), a selective mGluR1 antagonist, confirming previous findings that the LFS-induced LTD in LHb could be mediated by the activation of mGluR1^1,3^. In addition, either a CP-AMPAR antagonist (NASPM, 10 μM) or an NMDAR blocker (D-AP5, 10 μM) suppressed the LFS-induced LTD as much as LY3677385 did (Fig. 1a,c), suggesting that, in addition to the activation of mGluR1, the activation of NMDAR and CP-AMPAR lead to a long-lasting suppression of the excitatory transmission from the stria medullaris fibers into the LHb regions.

Despite synaptic NMDA signals not being noticed functionally in rat LHb^9^, the main components of NMDAR, GluN1, and GluN2A/B are known to exist meaningfully^11^. We thus investigated LFS-induced LTD under treatment with either an GluN2A- or an GluN2B-containing NMDAR antagonist to determine the specificity for receptor subtypes of NMDAR-dependent LTD in LHb (Fig. 1b,d). The LFS-induced LTDs were completely inhibited under treatment with NVP-AAM 077 (0.1 μM), an GluN2A-containing NMDAR antagonist and under ifenprodil (10 μM), GluN2B-containing NMDAR antagonist (Fig. 1b,d). All these results clearly show that the activation of GluN2A- or GluN2B- containing NMDARs leads to LTD induction triggered by LFS in LHb.

### Roles of CP-AMPAR on NMDAR-dependent LTD in LHb

**Figure 2:**
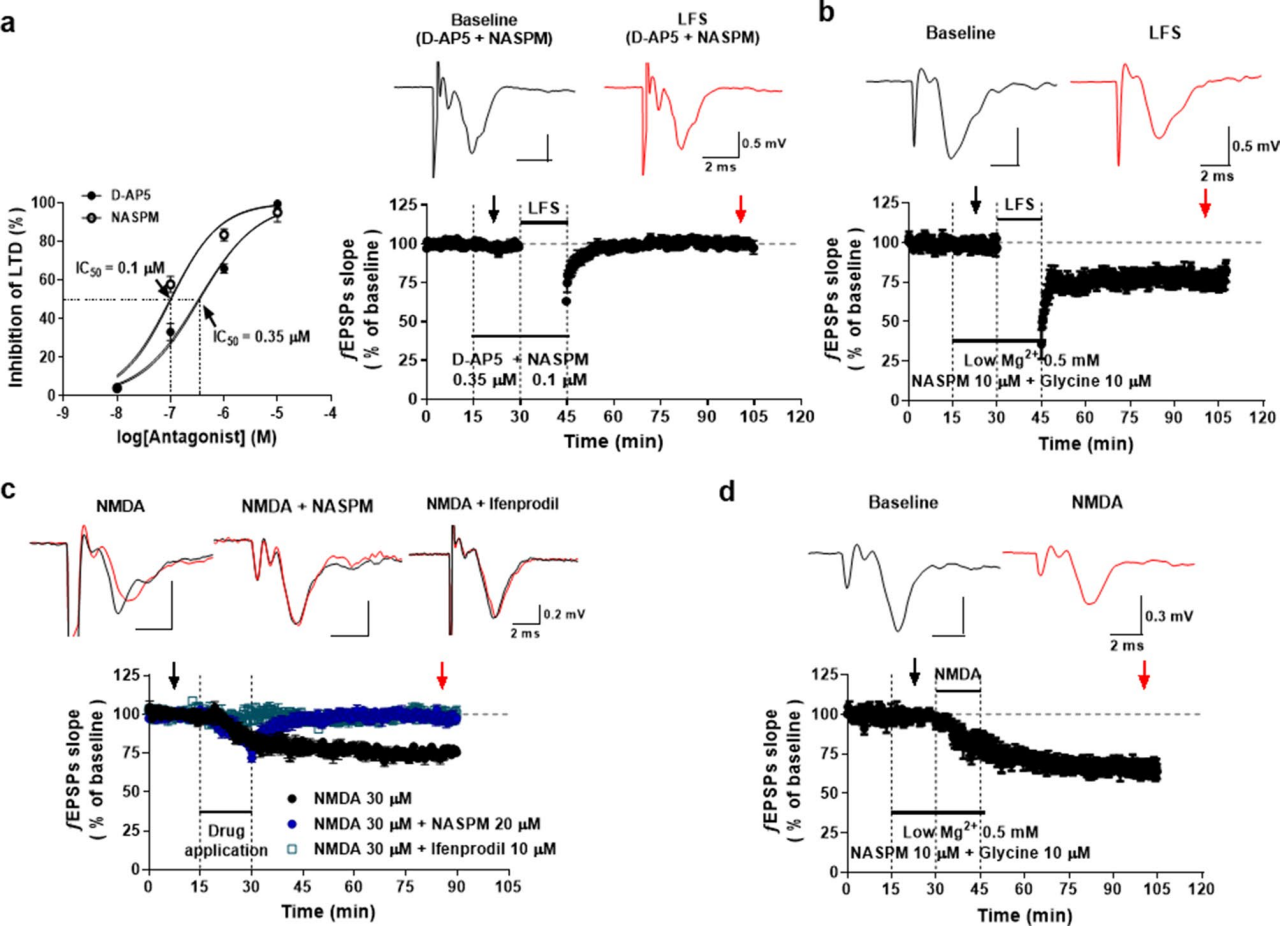
Roles of CP-AMPAR in NMDAR-dependent LTD. (**a**) *Left*; Dose response relationship of D-AP5 and NASPM on LFS-induced LTD (IC_50_ and Hill’s coefficient; D-AP5, 0.35 µM and 0.78 ± 0.1, *n* = 3; NASPM, 0.1 µM and 0.92 ± 0.1, *n* = 3). *Right*; Treatment with both D­AP5 (0.35 μM) and NASPM (0.1 μM) completely inhibited LTD induction in the LHb (97.4 ± 3.2%, *n* = 6). (**b**) NASPM failed to inhibit LFS-induced LTD in aCSF containing low Mg^2+^ (0.5 mM) and glycine (10 μM) (LFS-LTD, 77.9 ± 3.9%, *n* = 5). (**c**) Chemically NMDA-induced LTD was induced in the LHb after 15 min of treatment with NMDA (30 μM)(76.9 ± 2.2%, *n* = 6) and was completely inhibited by NASPM (20 μM)(96.3 ± 2.5%, *n* = 6) and by ifenprodil (10 μM)(98.6 ± 2.6%, *n* = 4). (**d**) NASPM did not block chemically NMDA-induced LTD (65.5 ± 5.6%, *n* = 5).

It has been generally reported that CP-AMPAR is concerned as a crucial regulator in NMDAR-dependent LTD^8^. We thus focused on the possible relation of CP-AMPAR with NMDAR-dependent LTD in LHb. First, as AMPAR and NMDAR-dependent LTD were not reported directly and systematically in the LHb, we investigated the actions of NASPM and D-AP5 on LFS-induced LTD. Both NASPM and D-AP5 suppressed the LFS-induced LTD in a concentration-dependent manner and their IC_50_ values were determined as 0.1 µM and 0.35 µM, respectively (Fig. 2a, *Left*). Such a dose–response relationship shows that the contribution of CP-AMPAR and NMDAR to LTD generation is authentic. We roughly predicted that if the actions of CP-AMPAR and NMDAR on LTD generation occur independently, the partial suppression effects of NASPM and D-AP5 would appear to be a simple arithmetic sum, while if there was interdependence, they would be larger or smaller than the arithmetic sum. We found that D-AP5 (0.35 µM), which partially suppressed the LFS-induced LTD (Fig. 2a, *Left*), inhibited the LTD completely when applied together with NASPM (0.1 µM) (Fig. 2a, *Right*). This strongly suggests an interdependence of CP-AMPAR and NMDAR activity in LTD generation.

Because CP-AMPAR is first introduced at an early stage of synaptic plasticity^8^, we hypothesized that CP-AMPAR might be involved in the initial process at NMDAR-dependent LTD as well. NMDAR activation requires unblocking of Mg^2+^ from the pore of NMDAR. The preemptive activation of CP-AMPAR by periodic electrical stimulation could unplug Mg^2+^ in NMDAR by inducing the membrane depolarization. To determine this issue, we investigated the LTD induction under a low Mg^2+^ concentration (0.5 mM) with high glycine concentration (10 μM). No block with NASPM (10 μM ) on the LTD generation was observed in the conditions under which the activity of NMDAR was maximized, implying that the NMDAR-induced LTD can be produced without CP-AMPAR activation as long as the NMDAR is off the Mg^2+^ pore block. In addition, NASPM failed to control LFS-induced LTD under the low Mg^2+^ and high glycine conditions (Fig. 2b).

To test this issue more, we examined the effect of NASPM on a chemically NMDA-induced LTD that was generated by applying NMDA (30 μM) alone for 15 min. The chemically NMDA-induced LTD was almost completely inhibited by the co-application of NASPM (20 μM) (Fig. 2c), indicating that the CP-AMPAR activity is required for the initiation of the NMDAR-induced LTD. It also completely blocked by ifenprodil, suggesting that chemically NMDA-induced LTD is NMDAR-dependent LTD (Fig. 2c). Moreover, NASPM (10 μM) also failed to chemically NMDA-induced LTD under the low Mg^2+^ and high glycine conditions (Fig. 2d). Taken together, we concluded that the CP-AMPAR activation may contribute to the initial process of NMDAR-dependent LTD by removing Mg^2+^ from NMDAR pore.

### Characteristics of NMDAR-dependent LTD in LHb

**Figure 3:**
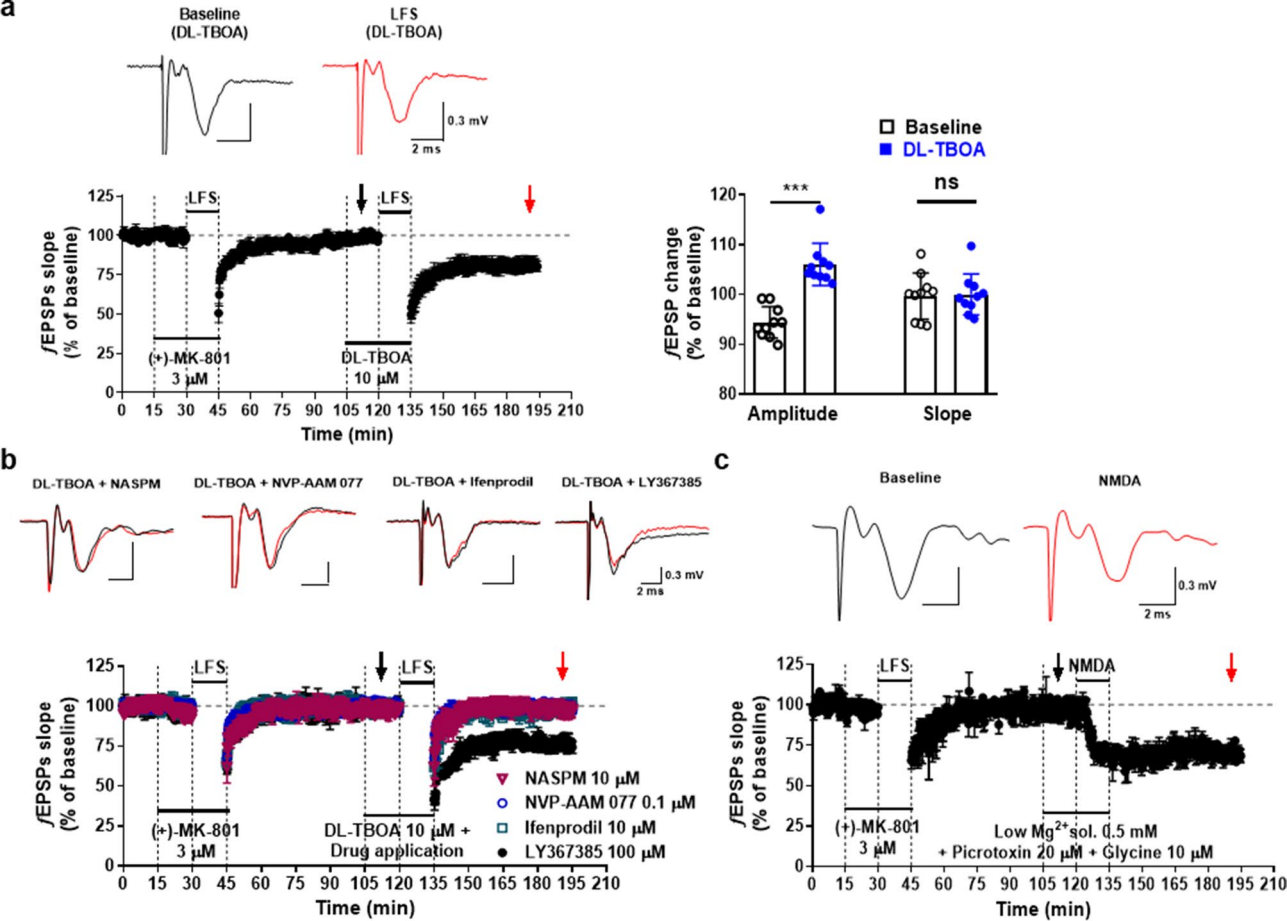
Function of CP-AMPAR and NMDAR for the induction of extrasynaptic LTD in the LHb. (**a**) *Left*; While the application of ( +)­MK-801 (3 μM) completely suppressed LFS-induced LTD (95.1 ± 1.4%, *n* = 6), LFS steadily induced extrasynaptic LTD after the application of DL-TBOA (10 μM) (81.0 ± 4.0%, *n* = 6). *Right*; After the treatment of DL-TBOA without LFS, the change of amplitude showed a significant potentiation five minutes after the drug application of DL-TBOA but did not lead to the change of fEPSP slope. (**b**) LFS-induced LTD completely inhibited by the co-application of DL-TBOA with NASPM (20 μM; 97.5 ± 3.3%, *n* = 3), NVP­AAM 077 (0.1 μM; 100.6 ± 1.7%, *n* = 5), ifenprodil (0.1 μM; 100.6 ± 1.7%, *n* = 5). However, the co­application of DL-TBOA with LY367385 (100 μM) did not show any inhibitory effects on LFS-induced LTD (74.1 ± 3.7%, *n* = 3). (**c**) After MK-801 exposure, chemically NMDA-induced LTD was generated in aCSF containing low Mg^2+^ (0.5 mM) and glycine (10 μM) (69.9 ± 2.0%, *n* = 2).

Recent microarray data^12^ indicate that the presence of GluN1 and GluN2B component of NMDAR in LHb is obvious, and our results confirmed that the expression of GluN2A as well as the expression of GluN2B was shown in LHb (Fig. S1b). Because GluN2A is found primarily within the synapses, wherease GluN2B is not, which is extrasynapses^20^, the following experiments were conducted to understand whether GluN2B NMDAR exists extrasynapse and, if so, whether its function induces synaptic plasticity. To block synaptic NMDAR in the LHb synapses, we applied LFS under the presence of MK-801, a well-known noncompetitive and irreversible blocker of NMDAR by exploiting the open channel blocking and “trapping” properties. No LTD was induced by LFS under MK-801 (3 μM) (Fig. 3a, left), confirming that the LFS-induced LTD in the LHb was synaptic NMDAR-dependent. No LTD was induced by another LFS given after more than 1 h of MK­801 washout (data not shown), showing that the NMDARs activated by the previous LFS were irreversibly and completely blocked by MK-801.

Moreover, to determine the role of extrasynaptic NMDAR on NMDA-dependent LTD, in the presence of the glutamate-uptake inhibitor DL-TBOA, we examined the effect of spillover and temporal summation of glutamate by blocking glutamate uptake. Under treatment with DL-TBOA (10 μM), LFS dramatically induced an extrasynaptic LTD (Fig. 3a, *left*). The ƒEPSP amplitude showed weak potentiation five minutes after the drug application of DL-TBOA without LFS but did not lead to the change of fEPSP slope (Fig. 3a, *right*). NASPM (20 μM) fully inhibited the LFS-induced LTD under DL-TBOA (Fig. 3b), showing that the activation of CP-AMPAR was required for the induction of extrasynaptic LTD. Those extrasynaptic LTDs was fully blocked by ifenprodil (10 μM) and NVP-AAM 077 (0.1 μM) (Fig. 3b), showing that the LTD induction could be mediated by the activation of GluN2A- and GluN2B-containing NMDAR expressed outside the synapses. It also indicates that both GluN2A- and GluN2B-containing NMDAR exist at the extrasynapse, as in the synapse, of an LHb neuron. However, unlike synaptic LTD, LTD under DL-TBOA did not inhibited in the treatment with LY367385 (100 μM) (Fig. 3b), showing that the activation of mGluR1 was not required for the induction of the extrasynaptic LTD. Furthermore, after MK-801 exposure, under low Mg^2+^ condition, we found chemically NMDA-induced LTD, suggesting that chemically NMDA-induced LTD also includes extrasynptic NMDAR-dependent LTD (Fig. 3c).

### Effect of acute stress on LTD generation in LHb

**Figure 4:**
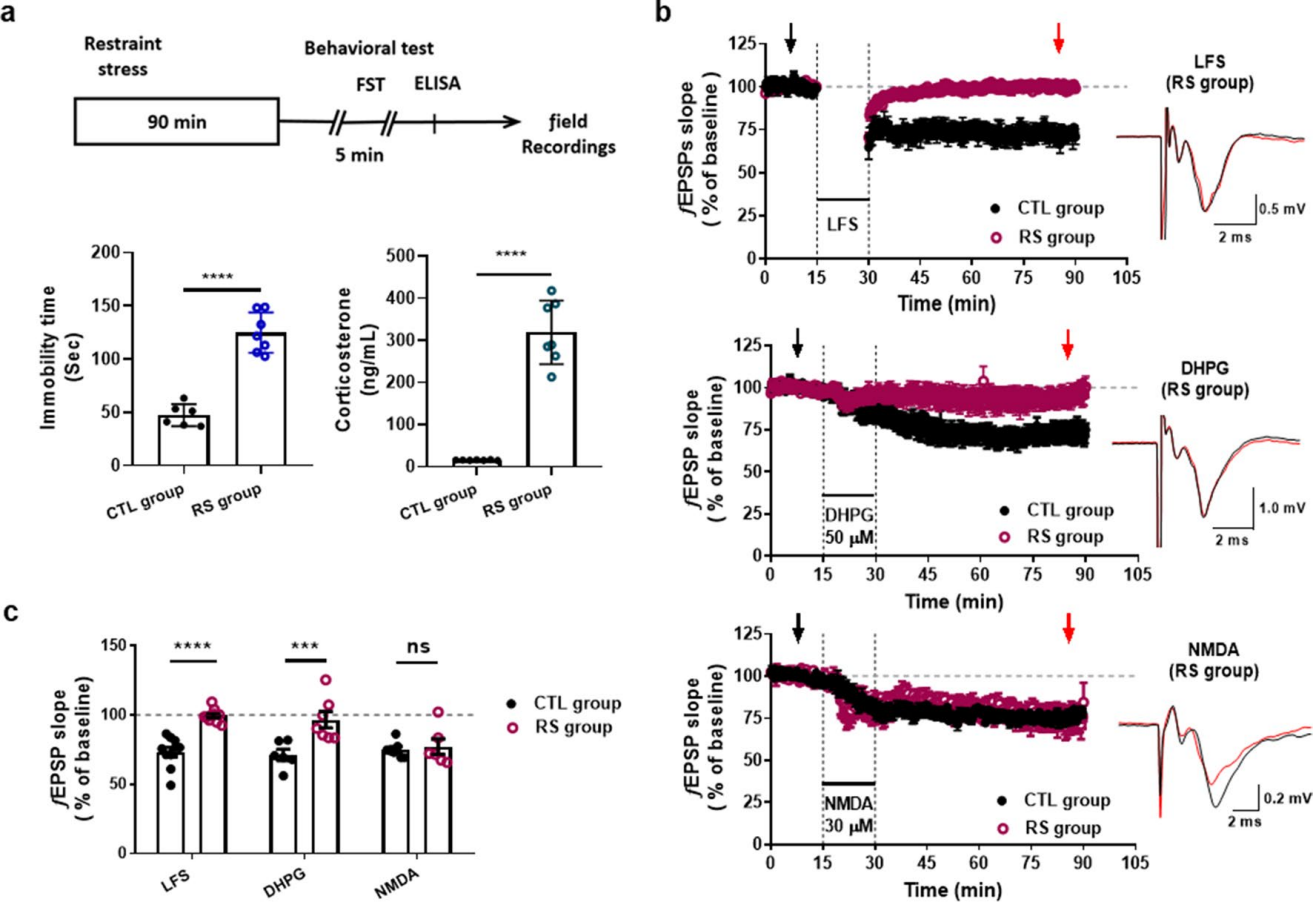
The effects of acute stress on NMDAR-dependent LTD. (**a**) *Top*, Restraint stress paradigm. Restraint stress was conducted for 90 min a day and then FST was carried out. Before extracellular recording, enzyme-linked immunosorbent assay (ELISA) was used to measure stress hormone corticosterone levels in the control (CTL) and acute restraint stress (RS) groups. *Bottom Left*, Immobility time (CTL group; 47.4 ± 4.2 s, *n* = 6, RS group; 124.8 ± 7.2 s, *n* = 7). *Bottom Right*, Level of corticosterone (CTL group, 15.6 ± 0.3 ng/mL, *n* = 7; RS group, 319.1 ± 28.5, *n* = 7). Static significance was determined using an unpaired *t*-test for comparisons between two groups (****P < 0.0001). (**b**) *Top*, LFS-LTD was completely blocked with rats exposed to restraint stress (99.6 ± 1.5%, *n* = 8). *Middle*, The acute stress-inhibited mGluR-dependent LTD which was induced by DHPG (50 μM) (CTL group, 73.6 ± 3.0%, *n* = 5; RS group, 96.8 ± 3.3%, *n* = 7). *Bottom,* Chemical LTD determined by NMDA (30 μM) was not affected and was well induced in the RS group (74.5 ± 7.2%, *n* = 6). (**c**) Summary histogram for differences in synaptic plasticity in the CTL group and RS group (two-way ANOVA; Holm-Sidak multiple comparisons, ****P < 0.0001, ***P = 0.0003, ns; *P* = 0.9722).

**Figure 5:**
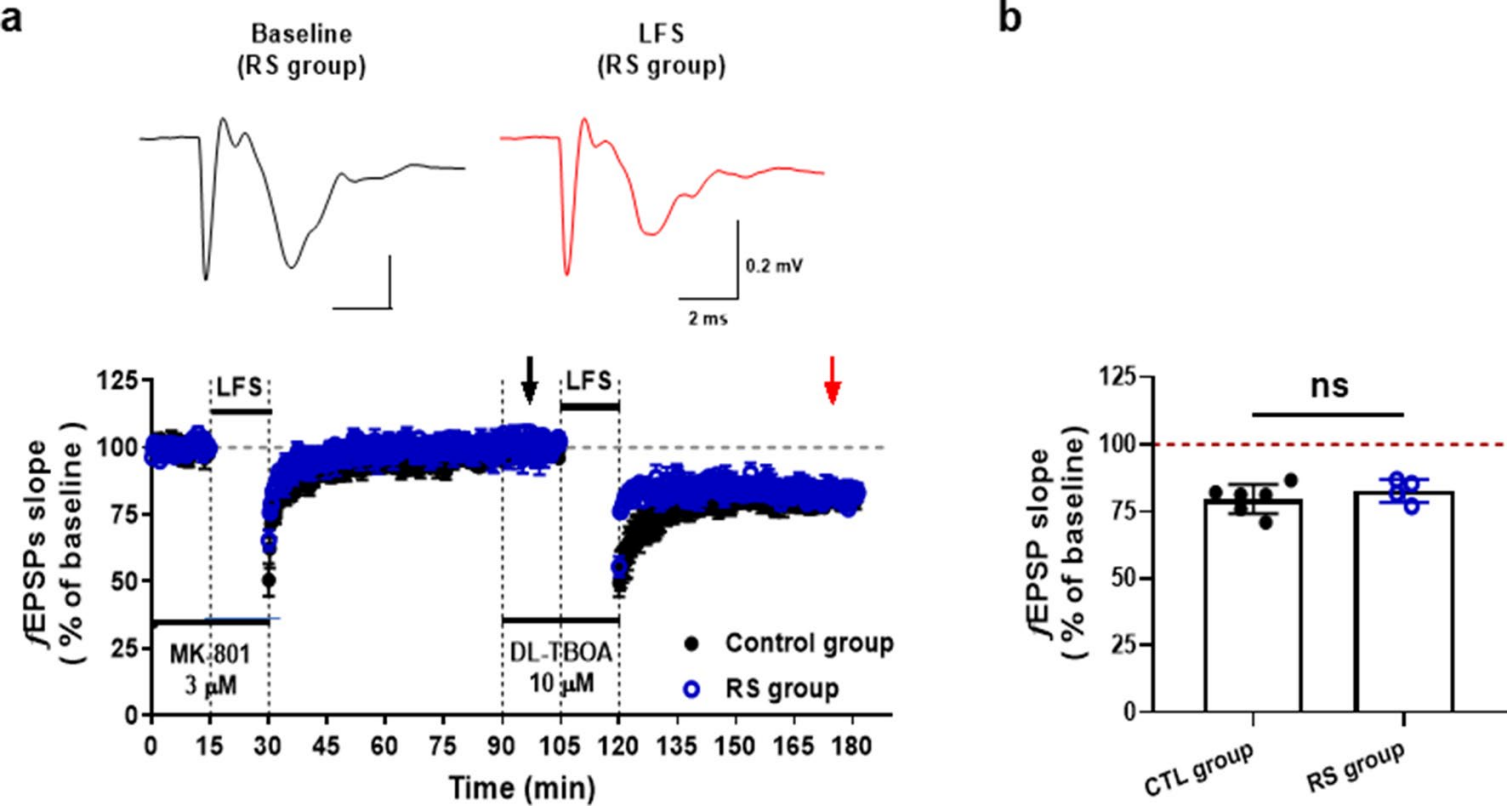
The effects of acute stress on extrasynaptic LTD. (**a**) In RS groups, LFS-induced LTD was generated when treated with DL-TBOA (10 μM). (**b**) Comparison of the differences in extrasynaptic LTD between control and restraint stress groups (unpaired *t-*test; CTL group, 79.7 ± 2.2%, *n* = 6; RS group, 78.8 ± 1.5%, *n* = 4, ns, *P* = 0.3857). Data are presented as mean ± standard error of the mean (S.E.M.).

To determine how the NMDAR-dependent LFS-LTD is changed by stress, we examined the effect of LTD in the LHb of restraint stressed rats. We found that the exposure to restraint stress for 90 min induced depressive-like behaviors and caused an excessive increase in corticosterone (Fig. 4a). The exposure of acute stress completely inhibited the LFS-LTD (Fig. 4b, *Top*), which is comparable to results of a previous study^3^. However, because our experimental techniques limit effective distinction between mGluR-dependent LTD and NMDAR-dependent LTD, it was difficult to determine which of them resulted in a reduction in the LTD due to stress. To circumvent this problem, we examined the effect of acute stress on both chemically DHPG-induced and NMDA-induced LTD (Fig. 4b). We found that no LTD was induced by DHPG (50 μM) under acute stress (Fig. 4b *Middle*, 4c), showing that mGluR-dependent LTD is highly sensitive to stress^3^. By contrast, the chemically NMDA (30 μM NMDA)-induced LTD was unaffected by stress (Fig. 4b *Bottom*, 4c), strongly suggesting that NMDAR-dependent LTDs are not affected by stress. We then hypothesized that the extrasynaptic LTD could not be affected by stress because our data showed that synaptic LTD was caused by either NMDAR or mGluR activity, whereas extrasynaptic LTD was only caused by NMDAR activity. In fact, the stress selectively prevented the generation of synaptic LTD (Fig. 5a). In the stress group, LFS did not induce synaptic LTD, whereas adding DL-TBOA led the LFS to induce LTD comparable to those in the control group (Fig. 5b). Taken together, our results suggest that the acute stress had a blocked effect on synaptic NMDAR-dependent LTD but left extrasynaptic NMDAR-dependent LTD intact.

## Discussion

In this study, we reported that various glutamate receptors are involved in the modulation of LFS-induced LTD in the LHb. First, NMDAR-dependent LTD in the LHb was specifically modulated by the NMDAR subtypes of GluN2A and GluN2B in the synaptic and extrasynaptic regions. The extrasynaptic NMDAR-dependent LTD had independent molecular mechanisms of group I mGluR. Second, we found that NMDAR and CP-AMPAR were dependently modulated in LTD for glutamatergic transmission. CP-AMPAR played a central role in activating NMDAR in the initial induction phase of NMDAR-dependent LTD. Third, acute stress exposure impaired synaptic NMDAR-dependent LTD but did not have an impact on extrasynaptic NMDAR-dependent LTD.

The projection of the excitatory synaptic response from the forebrain to the LHb containing CP-AMPAR displayed an inward rectification^9^. In Fig. 2, the activation of CP-AMPAR played an important role in NMDAR-dependent LTD and was involved in the long-lasting regulation of the excitatory synaptic response in the LHb. During the induction of NMDAR-dependent LTD in the hippocampal CA1 region, CP-AMPAR in the peri-synapse or the extrasynapse moves to the synapse and is involved in the activation of NMDAR, causing Ca^2+^ influx^8^. In our study, CP-AMPAR acted as an inducer to activate NMDAR and was necessary for NMDAR-dependent LTD.

The LTD generation was suppressed by MK-801, a selective synaptic NMDAR antagonist known as an open-channel blocker^21^, and LTD was not induced by LFS even after drug washout (*data not shown*). When synaptic NMDAR opened, MK-801 combined with the inside of synaptic NMDAR and blocked the flow of ions including Ca^2+^. During the suppression of NMDAR activation in the synaptic regions, DL-TBOA, a glutamate transporter (GluT) inhibitor, caused glutamate spillover in the synaptic cleft and was involved in the induction of extrasynaptic LTD. The extrasynaptic LTD was dependently modulated by the activation of GluN2A- and GluN2B-containing NMDAR as well (Fig. 3). Some studies reported that the GluN2A subtype is primarily expressed in the central portion of the synapse, whereas the GluN2B subtype is expressed in the peri-synaptic or extrasynaptic region^22,23^, while others reported that GluN2A- and GluN2B-containing NMDARs coexist in the extrasynaptic region^24^. Here, GluN2A- and GluN2B-containing NMDAR antagonists completely suppressed synaptic and extrasynaptic LFS-induced LTD (Figs. 1, 3). In immunohistochemistry assay, we also found that GluN2A and GluN2B subunits existed in LHb (Fig. S2a). Moreover, GluN2B did not affect the release probability of glutamate in the presynaptic sites (Fig. S2b). Therefore, NMDAR-dependent LTD in the LHb was specifically modulated by the NMDAR subtypes of GluN2A and GluN2B in the postsynaptic and extrasynaptic regions.

Generally, GluN2A-containing NMDAR has high open probability and leads to rapid inactivation in comparison with GluN2B-containing NMDAR. This property causes a relatively small amount of Ca^2+^ influx and facilitates AMPAR exocytosis. Meanwhile, GluN2B-containing NMDAR works opposite to GluN2A-containing NMDAR^25^. It has been reported that the GluN2A/GluN2B ratio determines the induction threshold of LTP and LTD^26^. When the synaptic GluN2A/GluN2B ratio is low following synaptic stimulation, LTP is more likely to occur rather than long-term depression (LTD). Conversely, when the GluN2A/GluN2B ratio is high, LTD is favored. The amount of Ca^2+^ influx from GluN2B­containing NMDAR, rather than from GluN2A-containing NMDAR, increases in low GluN2A/GluN2B synapses. However, LTD can be induced even in low GluN2A/GluN2B synapses. When the stimulation, such as LFS, is faintly transmitted in low GluN2A/GluN2B synapses, the amount of Ca^2+^ influx is insufficient for the activation of Ca^2+^/calmodulin-dependent protein kinase II (CaMKII). The insufficient influx of Ca^2+^ triggers the activation of calcineurin rather than CaMKII and induces LTD^26^. This means that even in low GluN2A/GluN2B synapses, the induction threshold of LTP and LTD can be changed depending on the integrated postsynaptic response. LFS-induced LTD is shown to be regulated by GluN2A- and GluN2B-containing NMDARs in the extrasynaptic region in the LHb. As animals grow up, GluN2B is moved from the synaptic region to the extrasynaptic region^13,14^. As this study used rats at postnatal days 17–24, it seemed highly probable that the LHb contained immature synapses and that GluN2A and GluN2B were mixed across the LHb^27^.

It has been suggested that the long-lasting inhibition of the excitatory synaptic response in the LHb may be closely related to stress and depression^1,3,9^. The molecular mechanism of LTD in the LHb is caused by the modulation of presynaptic neurons^1^, and the CBR1 signaling alteration mechanism caused by group I mGluR may be closely related to stress exposure^3^. Previous studies suggested that patients with major depression and animal models of depression showed decreased GluT in astrocytes^28,29,30^. The inhibition of GluT increases excitability in the LHb, which is likely to cause depressive-like behavior^18^. While the dysfunction of GluT in glial cells caused by stress may cause changes in depressive-like behavior, excessive glutamate spillover is likely to amplify LTD through the activation of extrasynaptic NMDAR (Fig. S3). The probability of glutamate release is increased in presynaptic neurons by stress^3,9^, and the amount of glutamate in the synaptic cleft may be excessively increased as GLT-1 dysfunction is caused in astrocytes^18^. Based on these findings, a glutamate increase in the synaptic cleft may inhibit synaptic LTD in the LHb but amplify extrasynaptic LTD (Fig. S3). As a result, extrasynaptic LTD can control neuronal activity in the LHb, which is excessively increased by stress, and play a key role in maintaining homeostasis.

## Supplementary information

Supplementary information.
