# Supplementary material for: NMDA receptor-dependent long-term depression in the lateral habenula: implications in physiology and depression
Source: Sci Rep. 2020 Oct 21;10:17921. doi: 10.1038/s41598-020-74496-w (PMC7578045; doi:10.1038/s41598-020-74496-w)
Supplement: Supplementary file 1 — Supplementary information. [file 41598_2020_74496_MOESM1_ESM.docx]

**Supporting Information**

**NMDA receptor-dependent long-term depression in the lateral habenula: implications in physiology and depression**

**Miseon Kang^1,3^, Jihyun Noh^*,2^, Jun-mo Chung^*,1^**

^1^ Department of Brain and Cognitive Sciences, Brain Disease Research Institute, Ewha Womans University, Seoul, Republic of Korea

^2^ Department of Science Education, College of Education, Dankook University, Yongin-si, Republic of Korea

^3^Emotion, cognition & behavior research group, Korea Brain Research Institute (KBRI), 61, Cheomdan-ro, Dong-gu, Daegu, 41062, South Korea

*Correspondence: [jihyun2@dankook.ac.kr](mailto:jihyun2@dankook.ac.kr) (J.N.), [jmchung@ewha.ac.kr](mailto:jmchung@ewha.ac.kr) (J.C.)

**Supporting Methods**

**Western blotting**

Standard western blotting procedures were followed^31^. Fifty micrograms of total protein were loaded in each lane. Membranes were washed three times with TBST every 20 minutes and detected with ECL^TM^ Prime western blotting detection reagent (GE Healthcare). Western blot images were produced using LAS-3000 and ChemiDoc™ XRS and analyzed using Image J Software. Primary antibodies against NR1, NR2A, and NR2B and those against β-actin and all secondary antibodies were from Cell Signaling. All primary antibodies were diluted at a ratio of 1:500, and secondary antibodies were diluted at a ratio of 1:1000.
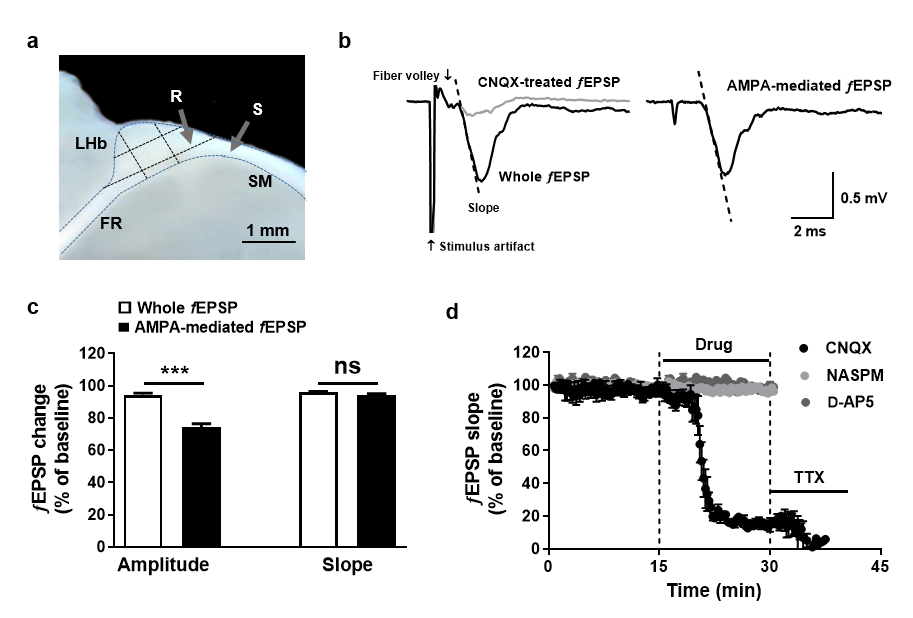
**Figure S1. Characteristics of ƒEPSPs in LHb**

(**a**) Photograph of a sagittal section containing the LHb. LHb, lateral habenula; FR, fasciculus retroflexus; SM, stria medullaris; S, stimulation electrode; R, recording electrode. Typical ƒEPSPs elicited by stimulating SM were obtained in the dorsal central LHb (▨). (**b**) Raw traces of a ƒEPSP recorded in the LHb. *Left*, black: Whole ƒEPSP trace without drug application; gray: CNQX-treated ƒEPSP trace. *Right*, AMPA-mediated ƒEPSP trace by subtracting the CNQX-treated ƒEPSP trace from the complete ƒEPSP trace. (**c**) Comparing the differences of amplitude and slope between the whole ƒEPSP and the AMPA-mediated ƒEPSP (paired *t-*test; ****P* < 0.001; Amplitude, *n* = 10; Slop, *n* = 30). Data are presented as mean ± standard error of the mean (S.E.M). (**d**) Normalized ƒEPSP slop plotted against time. After recording a stable baseline for about 15 min, receptor antagonist drugs were administered via the bath for 15 min. The ƒEPSP was only suppressed by CNQX but not by _D_-AP5 or NASPM. The remaining ƒEPSP was inhibited by TTX.

**
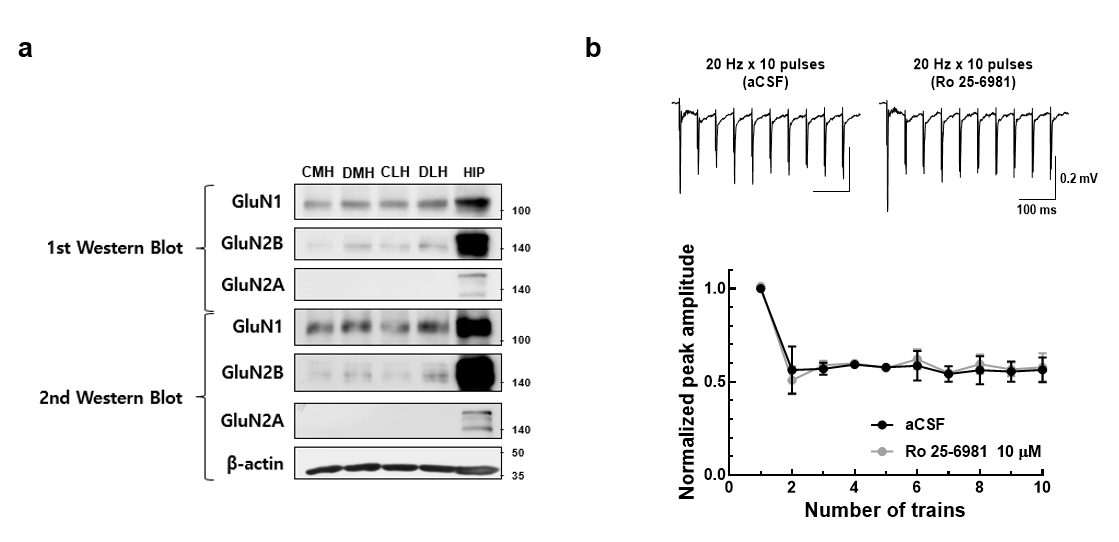
**

**Figure S2. NMDAR subtypes in the LHb**

(**a**) Protein expression of NMDAR subunits (GluN2A, GluN2B, and GluN1) in the LHb regions were detected by western blot analysis. At postnatal days (P) 17–21, we found that GluN1, GluN2A, and GluN2B protein was expressed. β-actin is shown as the control for comparison. CMH, control medial habenula; DMH, depression medial habenula; CLH, control lateral habenula; DLH, depression lateral habenula; HIP, hippocampus (**b**) Effects of GluN2B-containing NMDAR on the release probability of glutamate decrease in neurotransmitter showed the reduction of ƒEPSPs in response to stimulus trains at 20 Hz. Treatment with Ro 25-6981, GluN2B selective NMDAR antagonist, did not affect the decrease in ƒEPSP. The ƒEPSP sweep shows normal aCSF and Ro 25-6981 to describe the changes in ƒEPSP sweep.

**
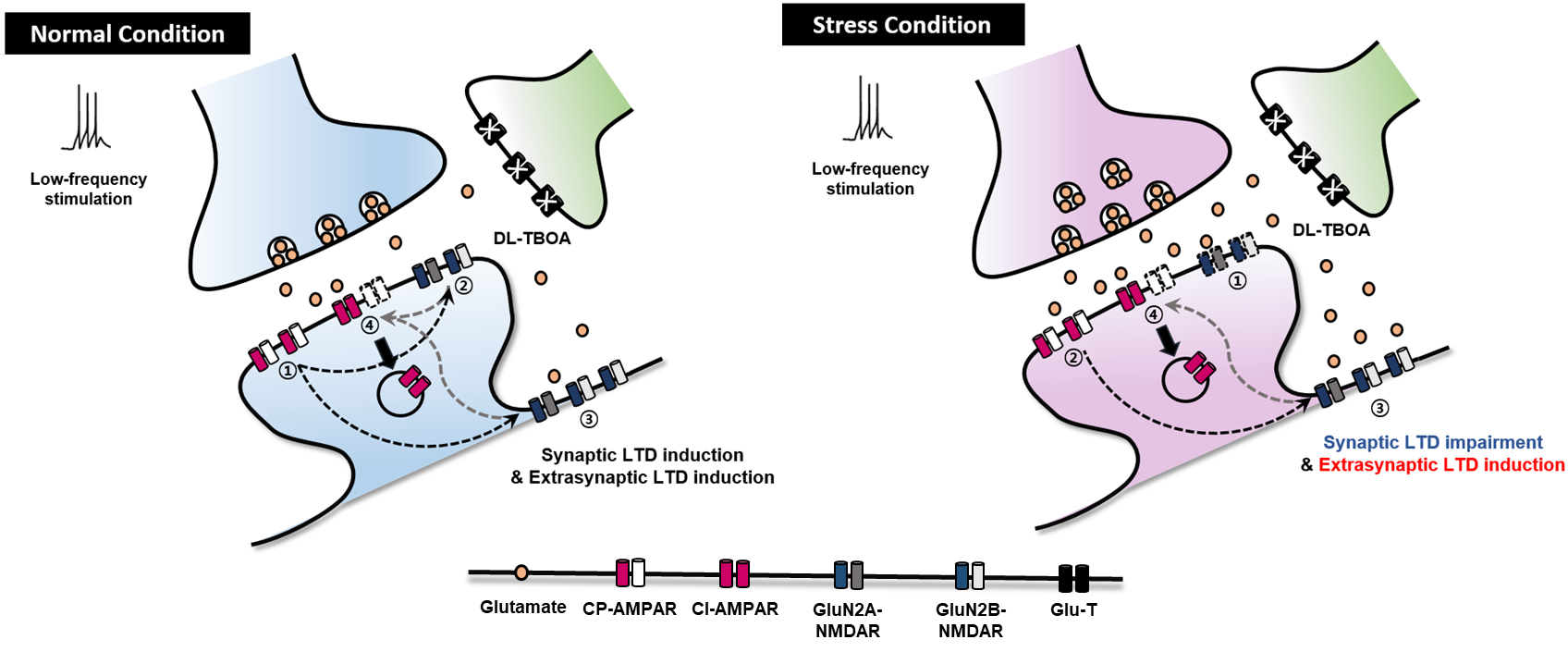
**

**Figure S3. The Role of NMDAR on LFS-induced LTD in the LHb region**

*Left,* **<Normal condition>** **①** NMDAR-dependent LTD requires the preferential activation of CP-AMPAR in the LHb. **②** Activation of the GluN2A- and GluN2B-containing NMDAR-induced LTD in the synaptic sites. **③** The glutamate spillover in the synaptic cleft causes activation of the GluN2A and GluN2B-containing NMDARs in the extrasynaptic sites ④ Therefore, NMDA receptor-dependent LTD could be induced in the synaptic and extrasynaptic sites.

*Right****,* <Stress condition>** **①** Glutamate increased by acute stress lead to LTD extinction of synaptic sites in the LHb region. **②,③** However, stress did not affect extrasynaptic LTD when DL-TBOA caused glutamate spillover in the extrasynaptic region. Thus, NMDARs are powerful regulators of excitation/inhibition (E/I) balance for neuronal activity in the LHb regions.
